# Emergence of Multidrug-Resistant *Escherichia coli* Producing CTX-M, MCR-1, and FosA in Retail Food From Egypt

**DOI:** 10.3389/fcimb.2021.681588

**Published:** 2021-07-13

**Authors:** Hazem Ramadan, Ahmed M. Soliman, Lari M. Hiott, Mohammed Elbediwi, Tiffanie A. Woodley, Marie A. Chattaway, Claire Jenkins, Jonathan G. Frye, Charlene R. Jackson

**Affiliations:** ^1^ Bacterial Epidemiology and Antimicrobial Resistance Research Unit, U.S. National Poultry Research Center, U.S. Department of Agriculture, Agricultural Research Service (USDA-ARS), Athens, GA, United States; ^2^ Hygiene and Zoonoses Department, Faculty of Veterinary Medicine, Mansoura University, Mansoura, Egypt; ^3^ Department of Microbiology and Immunology, Faculty of Pharmacy, Kafrelsheikh University, Kafr El-Sheikh, Egypt; ^4^ Animal Health Research Institute, Agriculture Research Center, Cairo, Egypt; ^5^ Institute of Preventive Veterinary Sciences & Department of Veterinary Medicine, College of Animal Sciences, Zhejiang University, Hangzhou, China; ^6^ Gastrointestinal Bacteria Reference Unit, Public Health England, London, United Kingdom

**Keywords:** *Escherichia coli*, retail food, whole genome sequencing, genetic context, *mcr-1*, *fos*A, cgMLST clustering

## Abstract

In this study, multidrug-resistant (MDR) *Escherichia coli* isolates from retail food and humans assigned into similar Multilocus Sequence Types (MLST) were analyzed using whole genome sequencing (WGS). *In silico* analysis of assembled sequences revealed the existence of multiple resistance genes among the examined *E. coli* isolates. Of the six CTX-M-producing isolates from retail food, *bla*_CTX-M-14_ was the prevalent variant identified (83.3%, 5/6). Two plasmid-mediated fosfomycin resistance genes, *fos*A3, and *fos*A4, were detected from retail food isolates (one each from chicken and beef), where *fos*A4 was identified in the chicken isolate 82CH that also carried the colistin resistance gene *mcr*-1. The *bla*_CTX-M-14_ and *fos*A genes in retail food isolates were located adjacent to insertion sequences IS*Ecp1* and IS*26*, respectively. Sequence analysis of the reconstructed *mcr-1* plasmid (p82CH) showed 96–97% identity to *mcr-1*-carrying IncI2 plasmids previously identified in human and food *E. coli* isolates from Egypt. Hierarchical clustering of core genome MLST (HierCC) revealed clustering of chicken isolate 82CH, co-harboring *mcr-1* and *fos*A4 genes, with a chicken *E. coli* isolate from China at the HC200 level (≤200 core genome allelic differences). As *E. coli* co-harboring *mcr-1* and *fos*A4 genes has only been recently reported, this study shows rapid spread of this genotype that shares similar genetic structures with regional and international *E. coli* lineages originating from both humans and food animals. Adopting WGS-based surveillance system is warranted to facilitate monitoring the international spread of MDR pathogens.

## Introduction

The emergence of multidrug resistance (MDR) in *Escherichia coli* has become a global health concern (Klemm et al., 2018). The continuous administration of antimicrobials in animals, either for treatment or prophylaxis and growth promotion purposes when fed to animals at sub-therapeutic doses, has led to the development of antimicrobial resistances that are able to disseminate to humans through the food chain (Pokharel et al., 2020; Van et al., 2020). The *bla*_CTX-M_ gene encodes an extended spectrum β-lactamase responsible for the hydrolysis of most β-lactams except cephamycins and carbapenems, and often co-exists with other genes conferring resistance to different antimicrobial classes such as aminoglycosides, tetracyclines, sulfonamides and fluroquinolones (Cantón et al., 2012; Lupo et al., 2018). Treatment of these bacterial infections is more complicated if resistance to colistin and fosfomycin is also present. These older antimicrobials have been reintroduced for the treatment of severe infections caused by MDR *E. coli* (Sherry and Howden, 2018; Karaiskos et al., 2019). Fosfomycin resistance in Gram-negative bacteria is frequently associated with the glutathione S-transferase-encoding gene *fos*A (Liu et al., 2020). Mobilized colistin resistance (*mcr*) gene acts by adding phosphoethanolamine to lipid A in lipopolysaccharides, modifying the bacterial cell wall and reducing susceptibility to colistin (Liu et al., 2016). The horizontal transfer of *bla*_CTX-M_ as well as *mcr* and *fos*A resistance genes is primarily linked to plasmids. However, other mobile genetic elements (MGEs) such as transposons and insertion sequences (IS) have also contributed to the transmission of these genes (Li et al., 2017; Zhang et al., 2019). Thus, studying the genetic context of these resistance genes would provide better understanding of the mechanisms responsible for their transmission.

The clonal diversity of *E. coli* has been determined using molecular typing techniques such as pulsed-field gel electrophoresis (PFGE) and multilocus sequence typing (MLST), to investigate and monitor the potential reservoirs of these isolates, their antimicrobial resistance and virulence traits (Nemoy et al., 2005). MLST assigned *E. coli* isolates into distinct sequence types (STs) based on the sequences of seven housekeeping genes (Tartof et al., 2005), that could indicate a possible epidemiological relationship if isolates from different hosts belong to the same STs (Ramadan et al., 2020b). The global spread of *E. coli* lineages, particularly those carrying emerging resistance genes, requires the implementation of whole genome sequencing (WGS)-based phylogenetic analyses for the discrimination of closely related isolates at higher resolution, compared to the seven-gene based MLST (Uelze et al., 2020; Zhou et al., 2020).

Previous studies from Egypt have reported the existence of antimicrobial-resistant *E. coli* isolates from retail food that could possibly disseminate to humans, yet there are few reports, to the best of our knowledge, that have analyzed the draft genome sequences of these isolates. Recently, we reported the circulation of shared MLST among the MDR *E. coli* isolated from retail food and humans in Egypt (Ramadan et al., 2020b). This study aimed to further characterize these isolates using WGS to explore the genetic environment of resistance genes they harbor, the associated plasmids, and to compare these isolates with the available *E. coli* genomes from Egypt, as well as the international lineages co-harboring colistin, *mcr*-1, and fosfomycin, *fos*A, resistance genes.

## Materials and Methods

### *E. coli* Isolates for Whole Genome Sequencing

Thirteen *E. coli* isolates from humans (n = 2) and retail food (n = 11; five isolates from whole chicken carcasses and six isolates from ground beef) were chosen from our previous study (Ramadan et al., 2020b) for further genetic characterization using WGS. *E. coli* isolates from i) food samples purchased from retail live chicken shops and supermarkets, and ii) stool of diarrheic patients admitted to Mansoura University Hospitals, were all recovered between April and July 2017 from Mansoura, Egypt. Isolates were selected to represent the shared eight STs belonging to four clonal complexes (CCs) identified from chicken and beef (ST224, ST1011, ST48/CC10, ST156/CC156, ST155/CC155 and ST58/CC155), human and chicken (ST10/CC10), and human and beef (ST226/CC226). The enrolled isolates were MDR as previously determined (Ramadan et al., 2020b) (**Table S1**). Antimicrobials and their minimum inhibitory concentrations (MICs) were to: ampicillin (≥32 μg/ml), amoxicillin/clavulanic acid (≥32/16 μg/ml), ceftriaxone (≥4 μg/ml), azithromycin (≥32 μg/ml), chloramphenicol (≥32 μg/ml), nalidixic acid (≥32 μg/ml), ciprofloxacin (≥4 μg/ml), sulfisoxazole (≥512 μg/ml), trimethoprim/sulfamethoxazole (≥4/76 μg/ml), tetracycline (≥16 μg/ml), gentamicin (≥16 μg/ml) and streptomycin (≥64 μg/ml).

### Whole Genome Sequencing and Analysis

Extraction of genomic DNA (gDNA) from *E. coli* isolates was performed using GenElute™ Bacterial Genomic DNA Kit (Sigma-Aldrich, St. Louis, MO). The quality check for gDNA was determined using a NanoDrop™ spectrophotometer, followed by measuring gDNA concentration on an Invitrogen Qubit 2.0 Fluorometer as instructed by the manufacturer (Life Technologies Inc., Carlsbad, CA). DNA libraries were constructed using Nextera™ XT DNA Preparation Kit and Nextera XT index primers (Illumina Inc., San Diego, CA) following the manufacturer’s protocol. Paired-end sequences of 2 × 250 bp length were then generated from DNA libraries using a 500-cycle MiSeq reagent kit version 2 (Illumina Inc., San Diego, CA) on an Illumina MiSeq system. Raw reads were assembled *de novo* into contigs using A5-miseq assembler (Coil et al., 2015). The assembled sequences were deposited into the National Center for Biotechnology Information (NCBI) under BioProject number PRJNA666443. The assembly statistics and accession numbers are available in **Table S2**. The assembled sequences of *E. coli* isolates were analyzed using ResFinder 4.1, PlasmidFinder 2.1, and SerotypeFinder 2.0 available at the Center for Genomic Epidemiology (CGE, https://cge.cbs.dtu.dk/services/) to identify resistance genes, plasmid replicons and serotypes, respectively. BLAST (https://blast.ncbi.nlm.nih.gov/Blast.cgi) search in combination with identification of insertion sequence (IS) elements using ISFinder (https://www-is.biotoul.fr/blast.php) illustrated the genetic environment of *bla*_CTX-M-14_, *bla*_CTX-M-15_, *bla*_CTX-M-55_, *fosA3*, *fosA4* and *mcr-1* genes among the examined isolates. Annotation of the assembled sequences was performed using DFAST (https://dfast.nig.ac.jp/), then genetic comparison was drawn using Easyfig (http://mjsull.github.io/Easyfig/) tool (Sullivan et al., 2011). Plasmid reconstruction from the WGS of isolates harboring *fos*A and *mcr-1* genes was performed using PLACNETw (Vielva et al., 2017). The reconstructed plasmids were then aligned to the NCBI database to determine the best plasmid match. Genetic comparison of the reconstructed plasmids and the retrieved plasmid sequences from NCBI was determined using BLAST Ring Image Generator (BRIG) tool (http://sourceforge.net/projects/brig).

### Phylogenetic Analysis of the Examined *E. coli* Isolates

To perform phylogenetic analysis, raw paired-end fastq reads of the thirteen isolates were imported into Enterobase (https://enterobase.warwick.ac.uk/), and compared to the available genomes of *E. coli* from Egypt updated December 4th, 2020, using single nucleotide polymorphisms (SNPs) and hierarchical clustering of core genome (cg) MLST (HierCC) based on 2,513 core genomic loci (Zhou et al., 2020). The chicken *E. coli* isolate 82CH co-harboring *mcr-1* and *fos*A4 was specifically compared to the global *E. coli* lineages, where 59 *E. coli* from NCBI, that co-harbored *mcr-1* and *fos*A genes and representing different sources were randomly selected for SNPs and HierCC in Enterobase. In both comparisons, isolates were mapped to the reference strain *E. coli* K-12 MG1655 for SNPs analysis, and designated to HC200 differing by ≤200 core genomic alleles. Metadata for the selected *E. coli* sequences from Enterobase and NCBI are listed in **Tables S3**, **S4**.

## Results and Discussion

### *In Silico* Analysis of Whole Genome Sequences of Retail Food and Human *E. coli*

ResFinder analysis showed the presence of several antimicrobial resistance genes among retail food (chicken and beef) and human isolates (**Table 1**). All isolates contained at least one β-lactamase gene (*bla*_TEM_, *bla*_CTX-M_, *bla*_OXA_ and/or *bla*_SHV_) except beef isolate 11M. The *bla*_CTX-M_ gene was identified in eight isolates: four from chicken (*bla*_CTX-M-14_), and two each from beef (*bla*_CTX-M-14_ and *bla*_CTX-M-55_) and human (*bla*_CTX-M-14_ and *bla*_CTX-M-15_) isolates. Of the six *bla*_CTX-M_ identified from retail food (chicken and beef) isolates, *bla*_CTX-M-14_ was the prevalent variant that constituted 83.3% (5/6). Recent studies reported high prevalence of *bla*_CTX-M-14_ among *E. coli* isolates from retail chicken meat from Japan (Hayashi et al., 2018) and feces of food animals from South Korea (Song et al., 2020). However, reports from Egypt have determined higher occurrence of other variants than *bla*_CTX-M-14_ in isolates from food animals such as *bla*_CTX-M-15_ in *E. coli* from rectal swabs of healthy cattle (Braun et al., 2016), *bla*_CTX-M-1_ from chicken and beef meat (Moawad et al., 2017) and *bla*_CTX-M-28_ from retail meat products (Sabala et al., 2021).

**Table 1:** Resistance genes, quinolone resistance-determining region (QRDR) point mutations, plasmid replicons, and serotypes of *Escherichia coli* from humans and retail food in Egypt.

Isolate ID/Source	Resistance genes	QRDR point mutations		Plasmid replicon (Inc) types	Serotypes
		*gyr*A	*par*C		
23ST/Human stool	*bla*_CTX-M-14_, *bla*_OXA-1_, *sul2*, *tet*B, *dfr*A14, *str*A, *mph*A, *aad*A1, *cat*A1, *mdf*A	S83L	ND	FII, FIA, FIB, Q1	O40:H4
4M/Ground beef	*bla*_OXA-10_, *bla*_SHV-12_, *bla*_TEM-1B_, *qnr*S1, *sul2*, *sul3*, *dfr*A17, *tet*A, *str*A, *str*B, *aad*A1, *aad*A5, *flo*R, arr-2, *cml*A1, *aac*(3)-IIa, *aph*(3’)-Ia, *mdf*A	ND	ND	FIB, FII, Q1	OND:H10
17M/Ground beef	*bla*_TEM-1B_, *sul1*, *sul3*, *dfr*A12, *tet*A, *aad*A1, *mph*A, *cml*A1, *mdf*A	S83L, D87N	S80I	FIA, FIB, FII, I1	O29:H9
82CH/Chicken carcass	*bla*_TEM-1B_, *bla*_CTX-M-14_, *mcr*-1, *fos*A4, *sul1*, *sul2*, *tet*A, *aad*A1, *str*A, *str*B, *aac*(3)-IIa, *flo*R, *mph*A, *mph*B, *lnu*(F), *dfr*A1, *mdf*A	S83L, D87N	S80I	FIA, FIB, FII, HI2, I2, Q1, B/O/K/Z	O109:H23
19M/Ground beef	*bla*_TEM-1B_, *bla*_CTX-M-14_, *sul1*, *sul2*, *sul3*, *dfr*A12, *tet*A, *aad*A1, *aad*A2, *aad*A22, *str*A, *str*B, *flo*R, *erm*B, *lnu*(F), *aph*(3’)-Ia, *aph*(4)-Ia, *aac*(3)-IV, *mdf*A	S83L, D87N	S80I	FIB, FII, X1, I1	O4:H16
4CH/Chicken carcass	*bla*_TEM-1B_, *bla*_CTX-M-14_, *sul1*, *sul2*, *dfr*A12, *tet*A, *aad*A1, *aad*A2, *flo*R, *mph*A, *lnu*(F), *aac*(3)-IIa, *aph*(4)-Ia, *aac*(3)-IV, *mdf*A	S83L, D87N	S80I	HI2, I1, p0111	O8:H16
21M/Ground beef	*bla*_TEM-1B_, *bla*_CTX-M-55_, *qnr*A1, *fos*A3, *aad*A2, *sul1*, *dfr*A12, *tet*A*, mph*A, *aac*(3)-IId, *mdf*A	S83V	S80I	FIB, FII, p0111	O40:H11
6CH/Chicken carcass	*bla*_TEM-1B_, *bla*_CTX-M-14_, *sul1*, *sul2*, *sul3*, *tet*A, *aad*A1, *aad*B, *aph*(3’)-Ia, *str*A, *str*B, *aac*(3)-IIa, *flo*R, *mph*A, *erm*, *lnu*(F), *cml*A1, *mdf*A	S83L, D87H	S80I	FIB, FII, HI2, I1, Q1, X1, p0111	O88:H16
20ST/Human stool	*bla*_CTX-M-15_, *bla*_TEM-1B_, *sul2*, *qnr*S1*, tet*A, *dfr*A14, *str*A, *str*B, *mdf*A	ND	ND	FIB	O101:H9
2M/Ground beef	*bla*_TEM-1B_, *qnr*S1, *str*A, *str*B, *aph*(3’)-Ia, *dfr*A14, *sul2*, *tet*A*, flo*R, *mdf*A	ND	ND	FIB, FII, X1, Q1, CoI	O22:H28
71CH/Chicken carcass	*bla*_OXA-10_, *flo*R, *qnr*VC, *aac*(6)-Ib-cr, *sul2*, *tet*A*, tet*B, *aad*A1, *aph*(3’)-Ia, *str*A, *str*B, *aac*(6’)-Ib3, *dfr*A14, *cml*A1, *mdf*A	S83L	ND	FIA, FIB, FII, X1	OND:H28
11M/Ground beef	*str*A, *str*B, *sul2*, *tet*A*, flo*R, *mdf*A	ND	ND	FIB, FII, X1, p0111	O86:H31
59CH/Chicken carcass	*bla*_TEM-1B_, *bla*_CTX-M-14_, *sul1*, *sul2*, *sul3*, *tet*A, *aad*A1, *aad*A2, *aph*(3’)-Ia, *str*A, *str*B, *aac*(3')-IIa, *flo*R, *mph*A, *lnu*(F), *dfr*A12, *cat*A1, *cml*A1, *mdf*A	S83L, D87H	S80I	FIB, FII, HI2, I1, p0111	O151/:H51

ND, not determined.

Resistome findings also revealed the presence of two plasmid-mediated fosfomycin resistance genes: *fos*A3 and *fos*A4 from beef 21M and chicken 82CH isolates, respectively (**Table 1**). Furthermore, the chicken *E. coli* isolate 82CH that carried *fos*A4 also co-harbored *mcr-1* responsible for colistin resistance. Recent reports from Egypt have shown the co-existence of *mcr-1* and *fos*A4 in *E. coli* isolated from chicken feces (Soliman et al., 2021) and retail chicken carcass (Sadek et al., 2021). This signifies the role of poultry as potential reservoirs for the persistence and dissemination of these antimicrobial resistances in Egypt.

Five *E. coli* isolates carried plasmid-mediated quinolone resistance (PMQR) genes; three isolates were from beef (2M, *qnr*S1; 4M, *qnr*S1; 21M, *qnr*A1) and one each from chicken (71CH, *qnr*VC) and human (20ST, *qnr*S1). Only in chicken isolate 71CH, acetyl transferase gene, *aac*(6)-Ib-cr, was co-harbored with *qnr*VC. The plasmid-mediated *qnr* genes encode pentapeptide repeat proteins that are responsible for quinolone resistance *via* protecting bacterial DNA gyrase and topoisomerase IV from quinolone inhibition (Strahilevitz et al., 2009). A recent study from Egypt reported high prevalence of *qnr* genes among Gram-negative clinical pathogens including *E. coli*, *Klebsiella pneumoniae*, *Serratia marcescens*, *Salmonella enterica* subsp. *arizonae* and *Pseudomonas aeruginosa*; 58.7% of these isolates carried at least one *qnr* gene (Khalifa et al., 2019). The existence of *qnr*-producing *E. coli* in retail chicken and beef meat as reported in recent studies from different countries including Egypt (Moawad et al., 2017), the United States (Tyson et al., 2019) and Philippines (Belotindos et al., 2021), poses a serious public health threat. The functional *qnr*VC gene had been commonly identified in Vibrionaceae family (Zhang et al., 2018) and in *P. aeruginosa* isolates in China (Lin et al., 2020) playing a crucial role in quinolone resistance. To our knowledge, this is the first report of *qnr*VC in an *E. coli* isolate globally. Chromosomal mutations to *gyr*A and *par*C were also detected, where *gyr*A S83L was the prevalent point mutation (8/13, 61.5%) followed by *par*C S80I (7/13, 53.8%) and *gyr*A D87N (4/13, 30.8%). All the examined thirteen isolates carried at least one resistance gene to the aminoglycosides [*str*A, *str*B, *aad*A, *aac*(3), *aph*(3’), *aph*(4), *aac*(6’)], folate pathway inhibitors (*sul*, *dfr*A), and tetracycline (*tet*A*, tet*B) (**Table 1**).

Our findings showed that plasmid incompatibility (Inc) types, IncFIB (12/13, 92.3%) and IncFII (11/13, 84.6%) were the predominant plasmid replicons identified from retail food and human *E. coli* (**Table 1**). This was in agreement with previous studies that determined higher frequencies of IncF replicon types among *E. coli* isolates, especially those that were MDR (Carattoli, 2009; Yang et al., 2015; Adenipekun et al., 2019). A recent study from Egypt demonstrated the existence of IncF replicon types in NDM-producing *E. coli* isolates from humans and dogs (Ramadan et al., 2020a). The wide dissemination of IncF replicons among *E. coli* isolates from different sources, and the association of resistance traits, e.g. genes encoding extended spectrum β-lactamases (ESBLs), carbapenemases, aminoglycoside-modifying enzymes, and PMQR genes, to this replicon type, could be responsible for possible interspecies dissemination of these plasmid-mediated resistance genes between humans and animals (Rozwandowicz et al., 2018; Adenipekun et al., 2019).

### Genetic Overview of *bla*_CTX-M_, *mcr-1* and *fos*A Genes and Associated Plasmids

**Figure 1:**
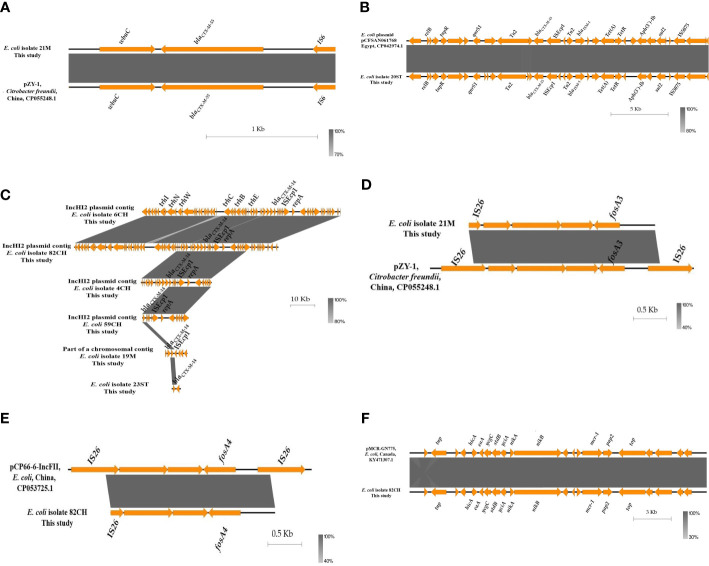
Schematic representation of the genetic environments of *bla*_CTX-M-55_ **(A)**, *bla*_CTX-M-15_ **(B)**, *bla*_CTX-M-14_ **(C)**, *fos*A3 **(D)**, *fos*A4 **(E)** and *mcr-1* **(F)** identified from the whole genome sequences of the examined *Escherichia coli* isolates from retail food and human. The figure was drawn using the EasyFig tool (http://mjsull.github.io/Easyfig/).

Genetic mapping of *bla*_CTX-M_ variants (*bla*_CTX-M-14_, *bla*_CTX-M-15_, and *bla*_CTX-M-55_), *mcr-1* and *fos*A has been determined as described in **Figure 1**. The *bla*_CTX-M-55_ identified in beef isolate 21M, was bracketed with insertion sequence (IS) IS*6* and *wbuC* (for a cupin fold metalloprotein), whereas *bla*_CTX-M-15_ in human isolate 20ST was located upstream to IS*Ecp1* (**Figures 1A, B**). The contigs carrying *bla*_CTX-M-55_ and *bla*_CTX-M-15_ exhibited 100% sequence similarity to the corresponding parts of the plasmid pZY-1 identified from *Citrobacter freundii* in China (accession no. CP055248.1) and plasmid pCFSAN061768 identified from *E. coli* in Egypt (accession no. CP042974.1), respectively. Six *E. coli* isolates, four from chicken (6CH, 82CH, 4CH, 59CH) and one each from beef (19M) and human (23ST) harbored *bla*_CTX-M-14_. In chicken and beef isolates, *bla*_CTX-M-14_ was located upstream to IS*Ecp1* similar to *bla*_CTX-M-15_ in human isolate 20ST. This was in concordance with previous studies reporting the association of IS*Ecp1* with different *bla*_CTX-M_ variants such as *bla*_CTX-M-1_, _-2_, _-9_, _-25_ (Rossolini et al., 2008), *bla*_CTX-M-15_ (Casella et al., 2018) and *bla*_CTX-M-14_ (Liao et al., 2015; Tadesse et al., 2018). The contigs carrying *bla*_CTX-M-14_ in the four chicken isolates showed high similarity to each other and to the backbone of the IncHI2 plasmid, while beef isolate 19M harbored a chromosomal *bla*_CTX-M-14_ (**Figure 1C**). This highlights the potential role of IS*Ecp1* in mobilization of *bla*_CTX-M-14_ regardless of whether the gene was located on plasmids or the chromosome (Zhao et al., 2020).

**Figure 2:**
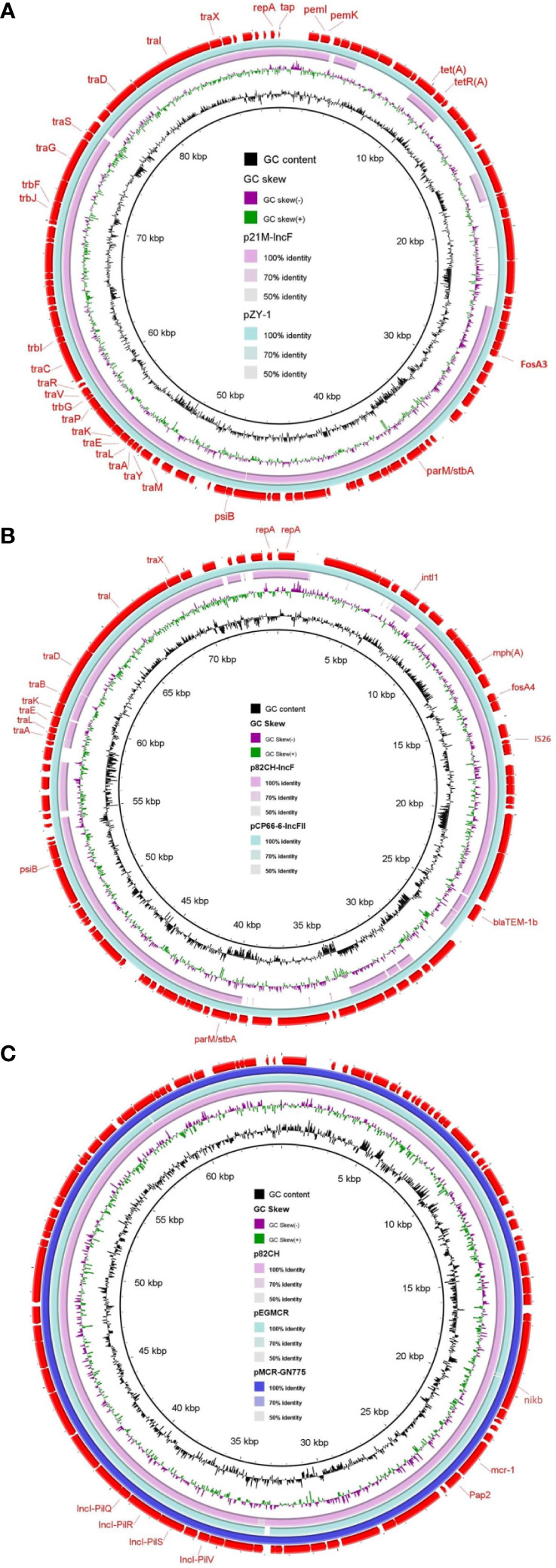
Comparative sequence analysis of *fosA3*-, *fosA4-* and *mcr-1-*carrying plasmids from the examined *Escherichia coli* isolates. Sequence comparison of reconstructed *fosA3* plasmid (p21M-IncF) from whole-genome sequence (WGS) of beef isolate 21M **(A)**. Sequence comparison of reconstructed *fosA4* plasmid (p82CH-IncF) from WGS of chicken isolate 82CH **(B)**. Sequence comparison of reconstructed *mcr-1* plasmid (p82CH) from WGS of chicken isolate 82CH **(C)**. The out-layer circle (red color) in **(A–C)** represents the reference plasmids used for sequence comparisons: pZY-1 plasmid (accession no. CP055248.1) **(A)**, pCP66-6-IncFII plasmid (accession no. CP053725.1) **(B)**, and pMCR-GN775 plasmid (accession no. KY471307.1) **(C)**. The figure was generated using BLAST Ring Image Generator (BRIG) tool (http://sourceforge.net/projects/brig).

Both variants of *fos*A, *fos*A3 in beef isolate 21M and *fos*A4 in chicken isolate 82CH, were located downstream to IS*26* belonging to the IS*6* family (**Figures 1D, E**), which might be involved in their mobilization. Sequences of *fos*A plasmids were recovered from WGS of beef (21M) and chicken (82CH) *E. coli* isolates using PLACNETw. The reconstructed *fos*A3 (p21M-IncF) and *fos*A4 (p82CH-IncF) plasmids showed approximately 72% sequence similarities to the entire IncFII plasmids pZY-1 (accession no. CP055248.1, size 89.6 kb) identified from *C. freundii* in China and pCP66-6-IncFII (accession no. CP053725.1, size 74.8 kb) identified from *E. coli* in China, respectively. The maintenance (*rep*A, replicase; *par*M/*stb*A, partition and stability; *psi*B, SOS inhibition) and transfer (*tra*) genes of p21M-IncF and p82CH-IncF plasmids were identical to the sequences of the corresponding genes of pZY-1 and pCP66-6-IncFII plasmids, respectively (**Figures 2A, B**). The existence of *fos*A in both isolates (21M and 82CH) adjacent to IS26 borne by IncFII plasmid, a widely known plasmid type for the dissemination of *fos*A worldwide (Benzerara et al., 2017), is alarming and could suggest retail chicken and beef as potential sources of this resistance gene to humans in Egypt. In our *mcr-1* isolate (82CH) from chicken carcass, the genetic context (*nikA*- *nikB* [encoding relaxase)- *mcr-1*- *pap2*- *top* (encoding a DNA topoisomerase III)] was detected without IS*Apl1*, a commonly associated IS with *mcr-1* (**Figure 1F**). A similar *mcr-1* context has been identified previously in *E. coli* from beef sausage in Egypt (Sadek et al., 2020). BLAST analysis of the reconstructed *mcr-1* plasmid (p82CH) from this study isolate showed 97% sequence similarity to the *mcr-1*-carrying IncI2 plasmid pMCR-GN775 (accession no. KY471307.1, size 64.6 kb) detected from an *E. coli* ST624 isolated from a Canadian patient with a history of hospitalization in Egypt (Tijet et al., 2017), and 96% similarity to IncI2 plasmid pEGMCR (accession no. MT499885.1, size ~64.1 kb) from a retail chicken isolate from Egypt (**Figure 2C**). IncI2 plasmid p82CH carried pilus and conjugative transfer proteins pilQ, pilR, pilS and pilV which are responsible for the conjugal transfer of that plasmid (Darphorn et al., 2021). This provides evidence that *mcr-1*-carrying IncI2 plasmid could be circulating among food animals and humans in Egypt.

### Phylogenetic Analysis of the Examined *E. coli* Using SNPs and HierCC

**Figure 3:**
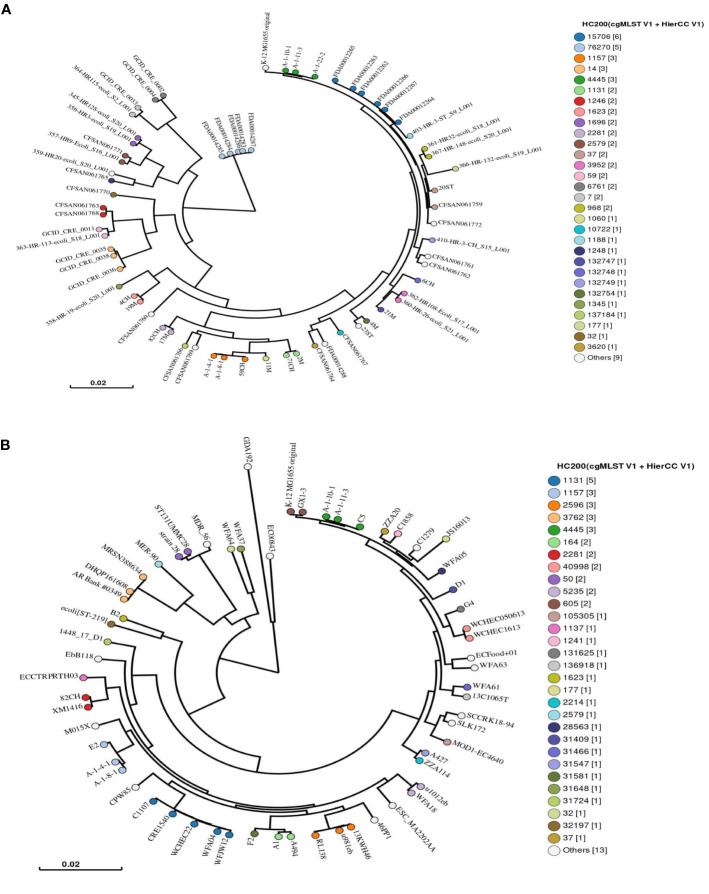
Single nucleotide polymorphisms (SNPs) and hierarchical clustering of cgMLST (HierCC) of the examined *Escherichia coli* isolates with the publicly available *E. coli* isolates from Egypt **(A)** in Enterobase (https://enterobase.warwick.ac.uk/). The chicken isolate 82CH co-harboring *mcr-1* and *fos*A4 genes was compared to 59 *E. coli* isolates from NCBI representing different sources using SNPs and HierCC analysis **(B)**. The legend shows the cgMLST HC200 that indicates allelic differences no more than 200 of 2,513 core genomic alleles among isolates.

SNPs and HierCC integrated into Enterobase were performed to investigate the phylogenetic relatedness of the examined *E. coli* isolates with publicly available *E. coli* genomes from Egypt (**Figure 3A** and **Table S3**). In Enterobase, HierCC designations have been set based on differences of 2,513 core genes among isolates with eleven designations: HC0, HC2, HC5, HC10, HC20, HC50, HC100, HC200, HC400, HC1100, HC2350; HC0 corresponds to indistinguishable isolates, HC1100 to ST lineage and HC2350 to *Escherichia* species (Zhou et al., 2020). Chicken isolate 59CH was clustered and assigned to similar HC200 (HC200_1157) with *E. coli* previously isolated from chicken feces (A-1-4-1 and A-1-8-1), which possibly explains the circulation of this clone among poultry in Egypt. When the chicken isolate 82CH was compared to the global *E. coli* co-harboring *mcr-1* and *fos*A, our isolate was clustered and shared a similar HC200 (HC200_2281) with an *E. coli* (XM1416) isolated from diseased broiler from China (**Figure 3B** and **Table S4**). This could indicate the wide circulation of this lineage co-harboring *mcr-1* and *fos*A4 across different continents that might be attributed to the global trade in food animals and food products (Ludden et al., 2020).

To conclude, this study reported the emergence of *bla*_CTX-M_, *fos*A and *mcr*-1 genes among retail food isolates in Egypt using WGS. Coupling our findings with recent reports from Egypt, we found that chicken could be the potential source for the emergence of *E. coli* co-harboring *mcr-1* and *fos*A4 genes. Moreover, comparative sequence analyses of *bla*_CTX-M_, *fos*A and *mcr*-1 genes and their associated plasmids among the examined *E. coli* from retail food, showed the existence of genetic features such as insertion sequences (IS) and certain plasmid Inc types, that are responsible for the mobilization and horizontal transfer of these genes. The global expansion of *E. coli* co-harboring *mcr-1* and *fos*A from different continents, requires the implementation of WGS for surveillance and control interventions.

## Author's Note

The mention of trade names or commercial products in this manuscript is solely for the purpose of providing specific information and does not imply recommendation or endorsement by the U.S. Department of Agriculture. USDA is an equal opportunity provider and employer.

## Data Availability Statement

The datasets presented in this study can be found in online repositories. The names of the repository/repositories and accession number(s) can be found below: https://www.ncbi.nlm.nih.gov/, PRJNA666443/.

## Author Contributions

HR and CRJ conceived and designed the study. HR performed whole genome sequencing of isolates, analyzed the data and wrote the original draft of the manuscript. AMS participated in data analysis and to the writing of the manuscript. LMH and TAW contributed to the laboratory work, whole genome sequencing and data analysis. ME contributed to genome analysis. CRJ and JGF secured funding and provided project administration. HR, AMS, ME, MAC, CJ, JGF and CRJ reviewed and edited the manuscript. All authors contributed to the article and approved the submitted version.

## Funding

This work has been funded by the U.S. Department of Agriculture (USDA) project 6040-32000-079-00-D. The Science, Technology and Innovation Fund Authority (STIFA) of Egypt (Short term fellowship, ID 25449) partially supported this work.

## Conflict of Interest

The authors declare that the research was conducted in the absence of any commercial or financial relationships that could be construed as a potential conflict of interest.
