# Supplementary material for: Emergence of Multidrug-Resistant Escherichia coli Producing CTX-M, MCR-1, and FosA in Retail Food From Egypt
Source: Front Cell Infect Microbiol. 2021 Jul 13;11:681588. doi: 10.3389/fcimb.2021.681588 (PMC8315045; doi:10.3389/fcimb.2021.681588)
Supplement: Supplementary file 1 [file DataSheet_1.docx]

Supplementary Material

**Table S1.** Source, sequence type (ST), clonal complex (CC) and minimum inhibitory concentration (MIC) of antimicrobials used for antimicrobial susceptibility testing of *Escherichia coli* isolates from retail food and humans.

| Isolate/ source | Sequence type (ST)/ clonal complex (CC) | MIC | | | | | | | | | | | | | |
| --- | --- | --- | --- | --- | --- | --- | --- | --- | --- | --- | --- | --- | --- | --- | --- |
|  |  | AM/CLA | AMP | AXO | FOX | NAL | CIP | STR | GEN | SXT | FIS | AZI | TET | CHL | MER |
| 23ST/ human stool | ST226/ CC226 | **= 32** | **> 32** | **> 64** | = 4 | **> 32** | = 0.5 | **> 64** | = 0.5 | **> 4** | **> 256** | **> 32** | **> 32** | **> 32** | <= 0.06 |
| 4M/ ground beef | ST226/ CC226 | = 16 | **> 32** | = 2 | = 4 | = 8 | = 0.5 | **> 64** | **> 16** | **> 4** | **> 256** | = 4 | **> 32** | **> 32** | <= 0.06 |
| 17M/ ground beef | ST224/ ND | = 8 | **> 32** | <= 0.25 | = 4 | **> 32** | **> 4** | = 32 | = 2 | **> 4** | **> 256** | **= 32** | **> 32** | **= 32** | <= 0.06 |
| 82CH/ chicken carcass | ST224/ ND | = 8 | **> 32** | **= 32** | = 2 | **> 32** | **> 4** | **> 64** | **> 16** | **> 4** | **> 256** | **> 32** | **> 32** | **> 32** | <= 0.06 |
| 19M/ ground beef | ST1011/ ND | = 16 | **> 32** | **= 32** | = 16 | **> 32** | **> 4** | **> 64** | **= 16** | **> 4** | **> 256** | **= 32** | **> 32** | **> 32** | <= 0.06 |
| 4CH/ chicken carcass | ST1011/ ND | = 8 | **> 32** | **= 64** | = 8 | **> 32** | **> 4** | **> 64** | **> 16** | **> 4** | **> 256** | **= 32** | **> 32** | **> 32** | <= 0.06 |
| 21M/ ground beef | ST48/ CC10 | = 8 | **> 32** | **= 64** | = 8 | **> 32** | **> 4** | **= 64** | **> 16** | **> 4** | **> 256** | **> 32** | **> 32** | = 8 | <= 0.06 |
| 6CH/ chicken carcass | ST48/ CC10 | = 16 | **> 32** | **= 64** | = 2 | **> 32** | **> 4** | **> 64** | **> 16** | = 0.25 | **> 256** | **> 32** | **> 32** | **> 32** | <= 0.06 |
| 20ST/ human stool | ST10/ CC10 | = 8 | **> 32** | **> 64** | = 4 | = 4 | = 0.5 | **> 64** | = 0.5 | **> 4** | **> 256** | = 8 | **> 32** | = 8 | <= 0.06 |
| 2M/ ground beef | ST156/ CC156 | = 8 | **> 32** | <= 0.25 | = 4 | = 8 | = 0.25 | **> 64** | = 0.5 | **> 4** | **> 256** | = 4 | **> 32** | **> 32** | <= 0.06 |
| 71CH/ chicken carcass | ST156/ CC156 | = 16 | **> 32** | = 0.5 | = 8 | **> 32** | = 2 | **> 64** | = 1 | **> 4** | **> 256** | = 4 | **> 32** | **> 32** | <= 0.06 |
| 11M/ ground beef | ST58/ CC155 | = 2 | = 2 | <= 0.25 | = 2 | = 2 | <= 0.015 | **> 64** | <= 0.25 | <= 0.12 | **> 256** | = 4 | **> 32** | **> 32** | <= 0.06 |
| 59CH/ chicken carcass | ST155/ CC155 | = 16 | **> 32** | **= 64** | = 16 | **> 32** | **> 4** | **> 64** | **= 16** | **> 4** | **> 256** | **= 32** | **> 32** | **> 32** | <= 0.06 |

Resistance to the antimicrobials is given in bold.

Interpretation was done according to the Clinical and Laboratory Standards Institute (CLSI) guideline (CLSI, 2017).

Amoxicillin/clavulanic acid, AM/CLA; Ampicillin, AMP; Azithromycin, AZI; Cefoxitin, FOX; Ceftriaxone, AXO; Chloramphenicol, CHL; Ciprofloxacin, CIP; Gentamicin, GEN; Meropenem, MERO; Nalidixic acid, NAL: Streptomycin, STR; Sulfisoxazole, FIS; Tetracycline, TET; Trimethoprim/sulfamethoxazole, SXT.

ND, not determined.

**References:** Clinical and Laboratory Standards Institute. Performance Standards for Antimicrobial Susceptibility Testing; Twenty-Seventh Informational Supplement M100-S27; CLSI: Wayne, PA, USA, 2017.

**Table S2.** Assembly statistics and Accession No. of the examined *Escherichia coli* isolates from retail food and humans.

| Isolate ID | Source of isolate | No. of Contigs | Genome Size (Mbp) | N50 (bp) | GC content (%) | Median coverage | Accession No. |
| --- | --- | --- | --- | --- | --- | --- | --- |
| 23ST | Human stool | 155 | 4.81 | 80632 | 50.5 | 85 | JACZFB000000000 |
| 4M | Ground beef | 108 | 4.87 | 151589 | 50.7 | 132 | JACZEZ000000000 |
| 17M | Ground beef | 88 | 5.15 | 218330 | 50.8 | 76 | JACZEX000000000 |
| 82CH | Chicken carcass | 244 | 5.73 | 168960 | 50.5 | 61 | JACZEQ000000000 |
| 19M | Ground beef | 110 | 5.31 | 322401 | 50.5 | 71 | JACZEW000000000 |
| 4CH | Chicken carcass | 132 | 5.4 | 238597 | 50.4 | 86 | JACZEU000000000 |
| 21M | Ground beef | 161 | 4.95 | 106386 | 50.7 | 68 | JACZEV000000000 |
| 6CH | Chicken carcass | 171 | 5.22 | 110198 | 50.3 | 80 | JACZET000000000 |
| 20ST | Human stool | 77 | 4.79 | 210298 | 50.8 | 74 | JACZFC000000000 |
| 2M | Ground beef | 221 | 5.18 | 63438 | 50.7 | 29 | JACZFA000000000 |
| 71CH | Chicken carcass | 147 | 5.28 | 134908 | 50.3 | 65 | JACZER000000000 |
| 11M | Ground beef | 110 | 5.28 | 156542 | 50.5 | 70 | JACZEY000000000 |
| 59CH | Chicken carcass | 148 | 5.41 | 153103 | 50.3 | 79 | JACZES000000000 |

**Table S3.** Metadata of the publicly available *Escherichia coli* genomes retrieved from Enterobase.

| Name | Bio Project ID | Sample ID | Source Niche | Source Type | Country | Collection Year |
| --- | --- | --- | --- | --- | --- | --- |
| CFSAN061769 | PRJNA230969 | SAMN06928087 | Food | Dairy | Egypt | 2016 |
| CFSAN061768 | PRJNA230969 | SAMN06928084 | Food | Dairy | Egypt | 2016 |
| CFSAN061767 | PRJNA230969 | SAMN06928085 | Food | Dairy | Egypt | 2016 |
| CFSAN061765 | PRJNA230969 | SAMN06909721 | Food | Dairy | Egypt | 2016 |
| CFSAN061764 | PRJNA230969 | SAMN06909722 | Food | Dairy | Egypt | 2016 |
| CFSAN061763 | PRJNA230969 | SAMN06909723 | Food | Dairy | Egypt | 2016 |
| CFSAN061762 | PRJNA230969 | SAMN06909724 | Food | Dairy | Egypt | 2016 |
| CFSAN061761 | PRJNA230969 | SAMN06909725 | Food | Dairy | Egypt | 2016 |
| CFSAN061760 | PRJNA230969 | SAMN06909726 | Food | Dairy | Egypt | 2016 |
| CFSAN061759 | PRJNA230969 | SAMN06909727 | Food | Dairy | Egypt | 2016 |
| CFSAN061771 | PRJNA230969 | SAMN06909729 | Food | Dairy | Egypt | 2016 |
| CFSAN061770 | PRJNA230969 | SAMN06928086 | Food | Dairy | Egypt | 2016 |
| CFSAN061772 | PRJNA230969 | SAMN06909728 | Food | Dairy | Egypt | 2016 |
| FDA00012263 | PRJNA230969 | SAMN07813266 | Environment | Plant | Egypt | 2017 |
| FDA00012267 | PRJNA230969 | SAMN07813269 | Environment | Plant | Egypt | 2017 |
| FDA00012266 | PRJNA230969 | SAMN07813262 | Environment | Plant | Egypt | 2017 |
| FDA00012265 | PRJNA230969 | SAMN07813268 | Environment | Plant | Egypt | 2017 |
| FDA00012264 | PRJNA230969 | SAMN07813267 | Environment | Plant | Egypt | 2017 |
| FDA00012262 | PRJNA230969 | SAMN07813265 | Environment | Plant | Egypt | 2017 |
| GCID_CRE_0033 | PRJNA508509 | SAMN10527298 | Human | Human | Egypt | ND |
| GCID_CRE_0002 | PRJNA508509 | SAMN10527282 | Human | Human | Egypt | ND |
| GCID_CRE_0004 | PRJNA508509 | SAMN10527283 | Human | Human | Egypt | ND |
| GCID_CRE_0038 | PRJNA508509 | SAMN10527302 | Human | Human | Egypt | ND |
| GCID_CRE_0036 | PRJNA508509 | SAMN10527300 | Human | Human | Egypt | ND |
| GCID_CRE_0035 | PRJNA508509 | SAMN10527299 | Human | Human | Egypt | ND |
| GCID_CRE_0011 | PRJNA508509 | SAMN10527288 | Human | Human | Egypt | ND |
| FDA00014285 | PRJNA230969 | SAMN11975263 | Companion Animal | ND* | Egypt | 2019 |
| FDA00014283 | PRJNA230969 | SAMN11975242 | Companion Animal | ND | Egypt | 2019 |
| FDA00014286 | PRJNA230969 | SAMN12035824 | Companion Animal | ND | Egypt | 2019 |
| FDA00014284 | PRJNA230969 | SAMN12036301 | Companion Animal | ND | Egypt | 2019 |
| FDA00014287 | PRJNA230969 | SAMN12036216 | Companion Animal | ND | Egypt | 2019 |
| FDA00014288 | PRJNA230969 | SAMN12036217 | Companion Animal | ND | Egypt | 2019 |
| 356-HR3-ecoli_S19_L001 | ND | ND | Companion Animal | Canine | Egypt | 2016 |
| 345-HR128-ecoli_S20_L001 | ND | ND | Human | Human | Egypt | 2016 |
| 357-HR9-Ecoli_S16_L001 | ND | ND | Companion Animal | Canine | Egypt | 2016 |
| 358-HR-19-ecoli_S20_L001 | ND | ND | Companion Animal | Feline | Egypt | 2016 |
| 359-HR20-ecoli_S20_L001 | ND | ND | Companion Animal | Canine | Egypt | 2016 |
| 361-HR32-ecoli_S18_L001 | ND | ND | Companion Animal | Feline | Egypt | 2016 |
| 363-HR-113-ecoli_S18_L001 | ND | ND | Companion Animal | Feline | Egypt | 2016 |
| 360-HR-26-ecoli_S21_L001 | ND | ND | Companion Animal | Feline | Egypt | 2016 |
| 364-HR115-ecoli_S2_L001 | ND | ND | Companion Animal | Feline | Egypt | 2016 |
| 362-HR108-Ecoli_S17_L001 | ND | ND | Companion Animal | Canine | Egypt | 2016 |
| 367-HR-148-ecoli_S20_L001 | ND | ND | Human | Human | Egypt | 2016 |
| 366-HR-132-ecoli_S19_L001 | ND | ND | Human | Human | Egypt | 2016 |
| 403-HR-3-ST_S9_L001 | ND | ND | Human | Human | Egypt | 2017 |
| 410-HR-3-CH_S15_L001 | ND | ND | Food | ND/Others | Egypt | 2017 |
| CFSAN061766 | PRJNA230969 | SAMN07248273 | Food | Dairy (Raw milk cheese) | Egypt | 2016 |
| A-1-4-1 | PRJNA661596 | SAMN16064908 | Poultry | Poultry | Egypt | 2019 |
| A-1-8-1 | PRJNA661596 | SAMN16064909 | Poultry | Poultry | Egypt | 2019 |
| A-1-10-1 | PRJNA661596 | SAMN16064910 | Poultry | Poultry | Egypt | 2019 |
| A-1-11-3 | PRJNA661596 | SAMN16064911 | Poultry | Poultry | Egypt | 2019 |
| A-1-22-2 | PRJNA661596 | SAMN16064912 | Poultry | Poultry | Egypt | 2019 |

ND*, Non determined.

**Table S4.** Metadata of the fifty-nine publicly available *Escherichia coli* co-harboring *mcr-1* and *fos*A genes.

| Name | Bio Project ID | Bio Sample ID | Source Niche | Source Type | Country | Collection Year |
| --- | --- | --- | --- | --- | --- | --- |
| MRSN388634 | PRJNA322756 | SAMN05173063 | Human | Human | United States | 2016 |
| RL138 | PRJEB13470 | SAMEA3980467 | ND/Others | ND/Others | ND* | ND |
| DHQP161608 | PRJNA335991 | SAMN05468123 | Human | Human rectal swab | United States | 2016 |
| CRE1540 | PRJNA360014 | SAMN06198938 | Human | Human rectal swab | Hong Kong | 2014 |
| WCHEC1613 | PRJNA275512 | SAMN04482604 | Water/River | Hospital sewage | Sichuan | 2015 |
| MDR_56 | PRJNA375745 | SAMN06344815 | Human | Human | United States | 2015 |
| ECFood+01 | PRJNA389557 | SAMN07203026 | Bovine | Beef | Thailand | 2016 |
| MER-90 | PRJNA398288 | SAMN07510255 | Human | Blood | Singapore | 2015 |
| ECCTRPRTH03 | PRJNA389557 | SAMN07450579 | Human | Human rectal swab | Thailand | 2017 |
| GX1-3 | PRJNA428878 | SAMN08323597 | Swine | Pig | China | 2015 |
| WCHEC22 | PRJNA418674 | SAMN06909177 | Human | Human | China | 2016 |
| WCHEC050613 | PRJNA418674 | SAMN06197014 | Water/River | Sewage | China | 2015 |
| SLK172 | PRJNA224116 | SAMN05853574 | Human | Human stool | China | 2015 |
| M015X | PRJNA293225 | SAMN10878258 | Human | Human stool | Sri Lanka | 2017 |
| ZZA114 | PRJNA408214 | SAMN07682694 | Poultry | Chicken anus swab | China | 2016 |
| XM1416 | PRJNA408214 | SAMN07682689 | Poultry | Sick broiler | China | 2016 |
| WFJW12 | PRJNA408214 | SAMN07682688 | Poultry | Chicken dung channel | China | 2016 |
| WFA37 | PRJNA408214 | SAMN07682671 | Poultry | Chicken feces | China | 2016 |
| WFA05 | PRJNA408214 | SAMN07682656 | Livestock | Sick pig lung | China | 2016 |
| JS16013 | PRJNA408214 | SAMN07682653 | Human | Patient Blood | China | 2016 |
| GDA192 | PRJNA408214 | SAMN07682645 | Swine | Pig anus swab | China | 2016 |
| CPW85 | PRJNA408214 | SAMN07682640 | Plant | Hospital effluent | China | 2016 |
| C1858 | PRJNA408214 | SAMN07682632 | Human | Patient hydatid fluid | ND | 2016 |
| C1279 | PRJNA408214 | SAMN07682628 | Human | Patient Urine | China | 2016 |
| WFA64 | PRJNA408214 | SAMN07682680 | Swine | Pig Liver | China | 2016 |
| WFA61 | PRJNA408214 | SAMN07682677 | Poultry | Chicken feces | China | 2016 |
| WFA18 | PRJNA408214 | SAMN07682663 | Poultry | Chicken Lung | China | 2016 |
| WFA04 | PRJNA408214 | SAMN07682655 | Swine | Pig Lung | China | 2016 |
| ZZA20 | PRJNA408214 | SAMN07682697 | Poultry | Chicken slaughterhouse splitting water | China | 2016 |
| WFA63 | PRJNA408214 | SAMN07682679 | Poultry | Chicken slaughterhouse plucked skin | China | 2016 |
| C1107 | PRJNA408214 | SAMN07682626 | Human | Patient Sputum | China | 2016 |
| A494 | PRJNA408214 | SAMN07682619 | Poultry | Chicken Liver | China | 2013 |
| A427 | PRJNA408214 | SAMN07682617 | Poultry | Chicken Anal swab | China | 2013 |
| 13KWH46 | PRJNA361032 | SAMN06219548 | Human | Human urine | Hong Kong | 2013 |
| 13C1065T | PRJNA361032 | SAMN06219550 | Poultry | Chicken feces | Hong Kong | 2013 |
| strain 28 | PRJNA342893 | SAMN05770810 | Human | Human | Singapore | 2014 |
| ESC_MA2202AA | PRJEB29576 | SAMEA5075912 | Human | Human urine | Switzerland | 2014 |
| ecoli[ST-219] | PRJNA551371 | SAMN12145232 | Human | Human urine | France | 2016 |
| MOD1-EC4640 | PRJNA230969 | SAMN12566307 | Environment | frozen coconut | Indonesia | 2018 |
| AR Bank #0349 | PRJNA554502 | SAMN12272266 | Human | Human | ND | ND |
| 1448_17_D1 | PRJEB34874 | SAMEA6086541 | Food | food | ND | ND |
| ST131UMMC28 | PRJNA488345 | SAMN10076849 | Human | Human blood | Malaysia | 2015 |
| G4 | PRJNA607307 | SAMN14127932 | Water/River | Sea water | ND | ND |
| F2 | PRJNA607307 | SAMN14127928 | Fish | Aquaculture | ND | ND |
| E2 | PRJNA607307 | SAMN14127922 | Poultry | Chicken | ND | ND |
| D1 | PRJNA607307 | SAMN14127916 | Water/River | Drinking water | ND | ND |
| C5 | PRJNA607307 | SAMN14127915 | Water/River | Irrigation water | ND | ND |
| B2 | PRJNA607307 | SAMN14127907 | ND/Others | Daipers | ND | ND |
| A1 | PRJNA607307 | SAMN14127890 | Water/River | River water | ND | ND |
| EbB118 | PRJEB37551 | SAMEA6658730 | Human | Human | Cambodia | 2017 |
| u981eb | PRJNA415804 | SAMN14923854 | Poultry | Skin sample (Gallus gallus domesticus) | United States | 2018 |
| SCCRK18-94 | PRJNA637815 | SAMN15158854 | Poultry | Chicken | China | 2018 |
| 46PP1 | PRJEB38313 | SAMEA6832605 | Swine | Sus scrofa | Thailand | 2018 |
| u1012eb | PRJNA415804 | SAMN15600339 | Poultry | Caeca content (Gallus gallus domesticus) | United States | 2018 |
| EC00843 | PRJNA475751 | SAMN16340178 | Human | Human | United States | 2019 |
| A-1-4-1 | PRJNA661596 | SAMN16064908 | Poultry | Poultry | Egypt | 2019 |
| A-1-8-1 | PRJNA661596 | SAMN16064909 | Poultry | Poultry | Egypt | 2019 |
| A-1-10-1 | PRJNA661596 | SAMN16064910 | Poultry | Poultry | Egypt | 2019 |
| A-1-11-3 | PRJNA661596 | SAMN16064911 | Poultry | Poultry | Egypt | 2019 |

ND*, Non determined.
